# What is the Youngest age Appropriate for Outpatient Surgery?

**Published:** 2009-02

**Authors:** Pramila Bajaj

**Affiliations:** Editor, IJA

Outpatient surgery accounts for a significant percentage of anaesthetics delivered in the United States and may be appropriate in infants for certain procedures. The most concerning adverse event after general anaesthesia in an infant is postoperative apnea.

There is little specific evidence of the risk of apnea in full term infants. There are facilities that feel comfortable performing outpatient surgeries if the infant is born at greater than 37 wk postconceptual age. However, other ambulatory centers prefer to wait untill the infant is 2-4 wk of age to ensure decreased physiologic jaundice, decreased pulmonary vascular resistance and to give time for the ductus arteriosus to close. As far as sudden infant death syndrome(SIDS) there is no evidence anaesthesia increases the risk^1^. However, if the patient has a sibling with a history of SIDS or if the mother has abused drugs in her pregnancy the risk increases many fold. The infants whose histories suggest a high risk for SIDS should be monitored closely for a longer perioperative period.

In the premature infant, apnea is more likely to occur as well as other airway complications such as atelectasis, aspiration pneumonia, stridor, and coughing with desaturation in infants undergoing inguinal herniorrhaphy^2^, The best evidence based data is found in Cote's^3^ combined analysis of 255 preterm infants undergoing inguinal herniorrhaphy under general anaesthesia. Apnea was defined as >15s without bradycardia or <15 s when accompanied by bradycardia. Apnea was strongly and inversely related to both gestational age and postconceptual age and to continuing apnea at home and anemia (<10 gm.dL^−1^). In the nonanemicchild with a gestational age of 32 wk and a postconceptual age of 56 wk, the probability of apnea was <1%. With a gestational age of 35 wk, a postconceptual age of 54 wk was the cutoff for apnea to be present in <1% of patients.

Caffeine has been shown to decrease the risk of apnea in preterm infants undergoing general anaesthesia. In 32 preterm infants (37-44 wk postconceptual age) who received caffeine 10 mg.kg^−1^ or placebo, the caffeine group had no postoperative bradycardia, prolonged apnea, periodic breathing or postoperative oxygen saturation <90%, whereas 81% of the patients in the control group had prolonged apnea at 4-6 h postoperatively^4^. A systematic review supported the evidence that caffeine reduces apnea risk^5^.

Spinal anaesthesia alone has been shown to have a lower incidence of postoperative apnea and bradycardia in former premature infants when compared to spinal plus sedation or general anaesthesia^6^. Also a decreased incidence of oxygen desaturation and bradycardia has been seen^7^. Central apne awas not reduced, so obstructive apnea may play a role with sedation or general anaesthesia^8^. Spinal anaesthesia may be indicated in high risk infants. Still the chance for cardiopulmonary events are increased in these infants, and the same postoperative monitoring as for generalan aesthesia is indicated^9^,^10^.

Patients <60wk postconceptual age for hernia repair had shorter times to extubation with no postoperative apnea after thiopental or halothane induction with desflurane maintenance than either halothane or sevoflurane for the entire anaesthetic^11^. Avoidance of opioids where possible and the use of regional anaesthetic techniques and nonopioid systemic analgesics such as acetaminophen and nonsteroidal antiinflammatory agents may decrease the risk of apnea.

The cutoff for outpatient surgery in infants born before 37 wk may be 50-52 wk postconceptual age as long as there is no anemia, prior apnea or coexisting disease. However, looking at the evidence based literature to decrease the risk of apneas to <1% patients should be greater than 54 wk postconceptual age without anemia, ongoing apnea or other significant medical problems. Postoperative monitoring ranges from 12-24h, including oxygen saturation, heart rate and impedance pneumography and whether the infants are apnea-free before discharge.

Caffeine or spinal anaesthesia may decrease the risk of apnea, but patients should not be discharged if they are not eligible for anaesthesia on an outpatient basis. Full-term infants are acceptable for outpatient procedures provided that they are otherwise healthy and the procedure is not likely to result in significant physiologic changes or postoperative pain requiring opioid medication and the anaesthetic is uneventful. Even in term infants some facilities will not allow outpatient surgery untill they are of 44-46 wk post conceptual age or require longer observation if younger, e.g., 4 h.

## References

[1] Steward DJ (1985). Is there risk of general ansthesia triggering SIDS? Possiblynot!. Anesthesiology.

[2] Steward DJ (1982). Preterm infants are more prone to complications following minor surgery than are term infants. Anesthesiology.

[3] Cote CJ, Zaslavsky A, Downes JJ (1995). Postoperative apnea in former preterm infants after inguinal herniorrhaphy. Anesthesiology.

[4] Welborn LG, Hannallah RS, Fink R (1989). High-dose caffeine suppresses postoperative apnea in former preterm infants. Anesthesiology.

[5] Henderson-Smart DJ, Steer P (2001). Prophylactic caffeine to prevent postoperative apnea following general anesthesia in preterm infants. Cochrane Database Syst Rev.

[6] Welborn LG, Rice LJ, Hannallah RS (1990). Postoperative apnea in former preterm infants. Prospective comparison of spinal and general anesthesia. Anesthesiology.

[7] Somri M, Gaitin L, Vaida S (1998). Postoperative outcome in high-risk infants undergoing herniorrhaphy: Comparison between spinal and general anesthesia. Anaesthesia.

[8] Krane EJ, Haberkern CM, Jacobson LE (195). Postoperative apnea, bradycardia, and oxygen desaturation in formerly premature infants: prospective comparison of spinal and general anesthesia. Anesth Analg.

[9] Frumiento C, Abajian JC, Vane DW (2000). Spinal anesthesia for preterm infants undergoing inguinal hernia repair. Arch Surg.

[10] Shenkman Z, Hopperstein D, Litmanowitz I (2002). Spinal anesthesia in 62 premature, former-premature or young infants: technical aspects and pitfalls. Can J Anaesth.

[11] O'Brien K, Robinson DN, Morton NS (1998). Induction and emergence in infants less than 60 weeks post-conceptual age: comparison of thiopental, halothane, sevoflurane and desflurane. Br J Anaesth.

